# Therapeutic Potential of Mulberry and Its Resilience to Abiotic and Biotic Stresses

**DOI:** 10.3390/ijms27072934

**Published:** 2026-03-24

**Authors:** Lanlan Feng, Rumeng Fu, Liming Bu

**Affiliations:** Henan Sericulture Research Institute, Henan Academy of Agricultural of Sciences, Zhengzhou 450002, China; furm@hnagri.org.cn

**Keywords:** mulberry, abiotic stress, biotic stress, economic value, medicinal properties

## Abstract

Mulberry is a plant species of significant economic value and is widely incorporated into various traditional medicinal formulations. Its multiple botanical parts (leaves, branches, fruits, seeds, and roots) possess both nutritional and therapeutic properties. Throughout its growth cycle, mulberry is exposed to a range of abiotic and biotic stresses. In response, the plant has evolved a suite of stress tolerance mechanisms, notably including the synthesis of diverse secondary metabolites. These metabolites, which encompass phenolic acids, flavonoids, and volatile aromatic compounds, exhibit pronounced pharmacological activities. This review systematically elucidates the roles of mulberry-derived phenolic compounds, alkaloids, and polysaccharides, which demonstrate a broad spectrum of biological effects, including antioxidant, antibacterial, antiviral, anticancer, anti-inflammatory, neuroprotective, anti-obesity, antidiabetic, and anti-enteritis activities. By integrating knowledge of mulberry’s adaptive mechanisms to abiotic and biotic stresses with the therapeutic functions of its extracts, this review aims to provide novel insights to guide future molecular breeding strategies and drug development efforts.

## 1. Introduction

Mulberry (hereinafter referred to as *Morus alba*) has long been cultivated in China for over 5000 years and holds a historically significant position as an economically vital tree species [1]. The widely cultivated mulberry, belonging to the genus *Morus* within the family Moraceae, is valued for its rich bioactive compounds and long history of use in traditional medicine. Among the multiple species within this genus, three are of particular agricultural and economic significance: the white mulberry (*Morus alba*), the red mulberry (*Morus rubra*), and the black mulberry (*Morus nigra*) [2]. Sericulture is a traditional agroeconomic system centered on the cultivation of mulberry trees for foliage used in rearing the silkworm (*Bombyx mori* L.), whose cocoons provide raw silk. Historically, silk trade facilitated early transcontinental exchange, fostering a proto-globalization between Europe and Asia via the Silk Road, established approximately 2000 years ago [3]. Chloroplast genome resequencing and de novo assembly of mulberry specimens from Samarkand, Uzbekistan, revealed that the white mulberry populations in this region originate from East Asia, specifically China [4]. In the contemporary textile industry, silk accounts for approximately 0.2% of the total global production value and is commercially produced in around 60 countries [5]. The leading producers include China, India, Uzbekistan, Vietnam, Thailand, and Brazil [6,7].

Beyond sericulture, mulberry fruits are consumed fresh or processed into beverages [8], jams [9], wines [10,11], vinegar [12], and food additives [13]. Additionally, mulberry leaves can be processed into herbal tea [14], used as an ingredient blended with wheat flour in traditional dumplings, noodles, steamed bun, or Indian paratha [15]. Both mulberry fruits and leaves can also serve as direct feed or processed feed supplements for livestock, including pigs [16], chickens [17,18], sheep [19,20], goats [21] and crucian carp [22]. Studies have demonstrated that dietary supplementation with mulberry-based ingredients enhances animal health performance [23].

In addition to their role as a nutritional resource for humans and livestock, mulberry was utilized in phytoremediation for their capacity to bioaccumulate heavy metal ions [24,25,26,27]. For example, riparian mulberry plantations in the Three Gorges Reservoir region (China) contribute to stormwater runoff mitigation, soil conservation, and phytoremediation of heavy metal pollutants [28]. Notably, the mulberry tree exhibits significant potential for the phytoremediation of cadmium contamination in contaminated soil [29,30]. Furthermore, specific bioactive compounds derived from mulberry have been formulated into cosmetic products for their anti-aging and skin-brightening properties [31,32].

Mulberry with its long cultivation history has been widely utilized in sericulture, food production, urban greening, and cosmetic applications. Nevertheless, comprehensive reviews systematically addressing its adaptive mechanisms and pharmacological potential under environmental stress are notably scarce. Accordingly, this article aims to critically synthesize current knowledge specifically in these two areas.

## 2. Environmental Stress Responses in Mulberry

**Figure 1:**
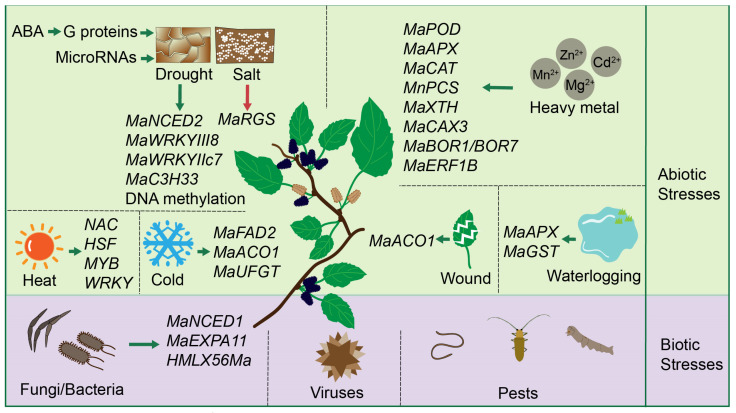
Abiotic and biotic stress factors in mulberry cultivation. Current research on abiotic stress in mulberry has predominantly concentrated on drought, salinity, heavy metal contamination, high temperature, freezing injury, mechanical damage, and waterlogging. Conversely, investigations into biotic stress have primarily addressed the major categories of fungal, bacterial, and viral diseases, as well as pest infestations. In the figure, the green and purple sections denote abiotic and biotic stresses, respectively. Green arrows represent positive regulation, while red arrows indicate negative regulation.

In recent years, plant growth has been increasingly challenged by the rising frequency of extreme weather events, extensive application of agrochemicals and microbial agents, and significant environmental pollution resulting from economic activities [33,34,35,36]. For economically vital crops, global environmental degradation and the spread of pathogenic invasions critically threaten agricultural productivity, thereby undermining global food security [37]. Current studies have demonstrated that in mulberry cultivation, synergistic interactions with arbuscular mycorrhizal fungi, intercropping with the leguminous plant alfalfa, or exogenous foliar spraying with 4-Chlorophenoxyacetic acid sodium salt (4-CPANa) can enhance mulberry quality [38,39,40]. However, as a key economic species supporting sericulture and medicinal applications, mulberry is likewise subject to a range of abiotic and biotic stresses throughout its growth cycle. Consequently, elucidating the molecular and physiological mechanisms underlying its stress adaptation has become an urgent research imperative. The following sections will systematically review the adaptive and regulatory responses of mulberry under adverse environmental conditions (Figure 1).

### 2.1. Abiotic Stresses

Heterotrimeric guanine nucleotide-binding proteins (G proteins), composed of α, β, and γ subunits, are known to play pivotal roles in plant responses to abiotic stresses. In mulberry, overexpression of the G-protein β subunit enhances drought tolerance in transgenic tobacco by modulating osmotic balance, antioxidative enzyme activities, and the expression of stress-responsive genes, suggesting a positive regulatory role for this subunit in plant adaptation to drought stress [41]. G-protein γ subunits MaGγ1 and MaGγ2 interact directly with core ABA (abscisic acid) signaling components MaABI1/2 and MaSnRK2.1/2.4, thereby enhancing ABA signaling transduction and ultimately regulating drought adaptation responses [42]. Furthermore, ectopic expression of mulberry G-protein subunits MaGβ, MaGγ1, and MaGγ2 in tobacco enhances drought and salt stress tolerance, likely through modulating reactive oxygen species detoxification [43]. MaRACK1 (the receptor for activated C kinase 1) functions as a negative regulator of drought and salt stress tolerance, with its overexpression reducing plant resilience through mechanisms independent of G-protein signaling and potentially mediated by interaction with fructose-1,6-bisphosphate aldolase [44]. Apart from that, under combined drought and salt stress, mulberry undergoes significant physiological alterations and exhibits distinct proteomic reprogramming, with sucrose metabolism playing a central role in its adaptive response across both leaves and roots [45]. *MaNCED2*, strongly induced by drought stress, catalyzes the cleavage of 9-cis-violaxanthin to produce xanthoxin, thereby contributing to ABA precursor biosynthesis in mulberry [46]. Mulberry enhances drought tolerance by activating pathways for proline and ABA biosynthesis, leading to significant accumulation of both compounds. The expression of *MaWRKYIII8* is up-regulated under drought conditions and in response to exogenous ABA, suggesting its potential involvement in the drought stress response of mulberry [47]. The mulberry transcription factor *MaWRKYIIc7* enhances drought tolerance by directly binding to W-box elements to upregulate stress-responsive genes (*MaNCED1* and *MaRD29A*), thereby promoting ABA-mediated signaling, improving ROS scavenging capacity, and reducing stomatal aperture [48]. MaC3H33, a nucleus-localized CCCH zinc finger protein in mulberry, is significantly induced by drought stress (exceeding 200-fold upregulation in stems), suggesting its potential role in regulating drought-responsive transcriptional networks [49]. Quantitative proteomics reveals that mulberry responds to drought stress by upregulating antioxidant enzymes, particularly glutathione peroxidase (GPX) isoforms, which enhance ROS scavenging to improve drought tolerance [50]. Mulberry enhances flavonoid biosynthesis through upregulation of phenylpropanoid pathway genes and specific glucosyltransferase activities, which contribute to antioxidant defense and osmotic adjustment, thereby supporting drought tolerance and offering potential for diversified utilization [51]. MicroRNAs, particularly conserved families such as mno-miR156, mno-miR172, and mno-miR396, play crucial roles in the drought stress response of mulberry by regulating a wide network of target genes, including transcription factors, through transcript cleavage and translational repression, thereby modulating diverse biological processes and metabolic pathways [52]. Beyond the direct regulatory roles of conserved microRNAs in drought response, differentially expressed long noncoding RNAs in mulberry form regulatory networks with differentially expressed messenger RNAs, primarily by modulating genes involved in secondary metabolite biosynthesis, and further recruit miRNAs to coordinate complex gene expression responses [53]. Another research showed that drought stress induces widespread DNA methylation changes in mulberry, characterized by a global increase in methylation predominantly at mCG sites, which likely regulates drought-responsive genes by targeting coding sequences and promoters, thereby modulating key biological processes involved in stress adaptation [54,55]. Interestingly, based on physiological, transcriptomic, and metabolomic analyses, the triploid mulberry cultivar (Shaansang-305) exhibits enhanced drought tolerance compared to its diploid progenitor, a trait associated with increased ABA content, upregulated expression of ABA-signaling and autophagy-related genes, and improved physiological adaptations including higher relative water content, SOD activity, and cuticular wax accumulation [56].

Mulberry responds to high-temperature stress (42 °C) through the differential expression of 703 genes, involving key metabolic pathways such as branched-chain amino acid degradation, starch/sucrose metabolism, and carotenoid biosynthesis, along with the activation of multiple transcription factor families (e.g., *NAC*, *HSF*, *MYB*, *WRKY*) to regulate heat adaptation mechanisms [57]. The *MaFAD2* gene, identified through GWAS as strongly associated with bud break timing, plays a key role in cold resistance by positively regulating dormancy maintenance in mulberry, with its expression levels directly correlating with delayed bud break and enhanced cold tolerance [58]. *MaACO1*, encoding ACC oxidase, is upregulated by both mechanical damage and low-temperature stress, and its elevated expression is closely associated with age-dependent tissue development, particularly in senescing leaves and pollinated reproductive structures of mulberry [59]. Another study showed that post-frost low temperature induces *UFGT* gene expression and enzyme activity in mulberry leaves, leading to the accumulation of flavonoid glycosides such as isoquercitrin and astragalin [60].

Under salt stress, mulberry seedlings maintain photosynthetic function by enhancing the activity and protein expression of both photosystems (PSII and PSI) and improving water use efficiency, whereas NaHCO_3_ stress leads to a comprehensive down-regulation of photosynthetic machinery and carbon assimilation primarily through non-stomatal limitations [61]. Mulberry germplasm exhibits significant genetic variation in salt tolerance at the seedling stage, with traits such as leaf area and biomass accumulation showing strong additive effects and serving as reliable morphological indices for evaluating and breeding elite, highly salt-tolerant genotypes [62]. The mulberry RGS protein (MaRGS) acts as a negative regulator of salt stress tolerance, as evidenced by enhanced sensitivity in overexpression lines and improved resilience in RNAi-silenced plants, potentially through mechanisms involving D-glucose and autophagy [63]. In addition, the salt-tolerant mulberry genotype S1 exhibited superior physiological adaptation compared to the salt-sensitive genotype ATP, as evidenced by better maintenance of biomass yield and cell membrane stability, higher accumulation of compatible solutes (proline and glycine betaine), and reduced lipid peroxidation [64].

Under waterlogging stress, mulberry initiates adaptive responses including the development of adventitious roots at the stem base, modulation of growth strategies, and repair of photosynthetic apparatus via enhanced non-photochemical quenching and thylakoid acidification mechanisms to mitigate physiological damage [65]. Furthermore, transcriptomic analysis reveals that mulberry tolerance to submergence stress is mediated by the upregulation of genes involved in ROS homeostasis (e.g., ascorbate peroxidase and glutathione S-transferase), energy metabolism (glycolysis, fermentation, and TCA cycle), and phytohormone signaling (ethylene, cytokinin, and abscisic acid) [66].

Mulberry demonstrates strong tolerance to heavy metal co-contamination by effectively accumulating Cd, Pb, and Zn in its roots, maintaining stable antioxidant enzyme activities, reducing lipid peroxidation, and enhancing rhizosphere microbial diversity, particularly benefiting arbuscular mycorrhizal fungal communities [67]. Moreover, another study reveals mulberry exhibits high tolerance to Cd stress through coordinated physiological adaptations, including maintained photosynthesis, enhanced antioxidant enzyme activities (POD, APX, CAT), regulated macronutrient homeostasis, and effective Cd accumulation in aboveground tissues, supporting its potential for phytoremediation of Cd-contaminated soils [29]. Calcium has been proved to antagonizes cadmium toxicity in mulberry by suppressing Cd influx and accumulation through competitive inhibition at transport sites, enhancing antioxidant defenses, and stabilizing Ca^2+^ signaling pathways, thereby mitigating oxidative stress and growth inhibition [68]. Overexpression of *MnPCS* (*Morus notabilis* phytochelatin synthase) enhances Zn and Cd tolerance in transgenic *Arabidopsis* and tobacco by promoting heavy metal accumulation and detoxification, with *MnPCS1* playing a more critical role in cadmium detoxification than *MnPCS2* [69]. Based on transcriptomic and RT-qPCR analyses of mulberry under excess Zn stress, a tissue-specific regulation of lignin biosynthesis was observed, characterized by the enhanced expression of lignin biosynthetic genes in lignified organs and their suppression in leaves, suggesting that modulating lignin deposition is a key physiological response to heavy metal stress in mulberry [70]. Under Mg imbalance, mulberry modulates antioxidant enzyme activities and non-enzymatic contents, disrupts chloroplast and mitochondrial ultrastructure, and alters the expression of genes involved in photosynthesis, chlorophyll degradation, antioxidant defense, and carbohydrate/energy metabolism, revealing a complex physiological and molecular regulatory network underlying magnesium stress tolerance [71]. Mulberry *MaXTH* genes, encoding xyloglucan-modifying enzymes, play a critical role in Mg stress adaptation by regulating cell wall remodeling, carbohydrate metabolism, and stress-responsive signaling pathways [72]. Under Mn stress, another study indicated that mulberry activates a coordinated response involving enhanced antioxidant defenses, modulation of cell wall composition, and transcriptional reprogramming of genes related to Mn transport and detoxification, with functional validation highlighting the key role of *MaCAX3* (encoding a cation/proton exchanger) in Mn homeostasis [73,74]. Boron deficiency in mulberry manifests as upward cupping of young leaves, followed by lenticel-like cracks along major veins, petioles, and stems, accompanied by reduced boron and chloroplastic pigment contents, elevated tissue Fe, Mn, and Zn concentrations, and increased leaf water potential and relative water content [75]. Boron deficiency and toxicity in mulberry trigger extensive transcriptomic reprogramming, involving coordinated alterations in key aquaporins, high-affinity boron transporters (BOR1/BOR7), antioxidants, photosynthesis-related genes, and hormone signaling regulators such as ERF1B [76]. Copper excess in mulberry plants induces root damage, accelerates senescence in older leaves, and triggers antioxidant responses while disturbing the cellular redox environment in young leaves [77].

### 2.2. Biotic Stresses

*Lasiodiplodia theobromae* isolates infecting mulberry in Guangxi exhibit significant genetic diversity and population structuring closely associated with their geographical origins [78]. In mulberry brown spot disease, six Colletotrichum species were identified, among which *C. fioriniae* is the primary causal agent of anthracnose, with *C. brevisporum*, *C. karstii*, and *C. kahawae* subsp. *ciggaro* acting as secondary pathogens, while *C. fructicola* and *C. cliviae* are non-pathogenic to mulberry [79]. Morphological and multi-gene phylogenetic analyses established *Boeremia maritima* as the causal agent of leaf spot disease on mulberry in northern Thailand, marking its first report from both this host and a terrestrial ecosystem [80]. *Euzophera semifuneralis*, the American plum borer, poses a dual threat to mulberry by directly infesting the tree and potentially vectoring the pathogenic fungus *Ceratocystis fimbriata*, which could have significant implications for plant health [81].

In 1999, a severe bacterial blight caused by *Pseudomonas syringae* pv. mori was found to affect white mulberry in the eastern Anatolia region of Turkey, with nearly 100% incidence in the surveyed regions. The pathogen was isolated, characterized, and its pathogenicity confirmed through Koch’s postulates, marking the first report of this disease with high incidence in that specific geographic area [82]. The plant-pathogenic bacterium *Enterobacter mori* is the causative agent of bacterial wilt disease in mulberry [83]. *Enterobacter cloacae* was identified as the causative agent of a novel bacterial wilt disease in mulberry, characterized by progressive leaf wilting from lower to upper foliage and distinct vascular discoloration [84]. *Xylella fastidiosa*, the causal agent of mulberry leaf scorch (MLS), was isolated and characterized from infected white mulberry trees in southern California, with strains showing genetic similarity to a previously characterized MLS strain from Virginia and exhibiting host specificity by failing to infect grapevines or oleanders [85]. Infection by mulberry dwarf phytoplasma triggers significant proteomic alterations, particularly the degradation of key photosynthetic proteins (e.g., rubisco large subunit and activase), which disrupts chloroplast integrity and carbon assimilation, ultimately leading to the characteristic physiological and morphological stress symptoms in mulberry [86].

Viral diseases represent a significant constraint on the productivity and commercial value of mulberry. A novel geminivirus, tentatively named mulberry crinkle leaf virus isolate Jiangsu (MCLV-js), was identified in mulberry plants exhibiting crinkle leaf symptoms and characterized by a distinct genomic organization featuring five contiguous GAAAAA repeats upstream of ORF1, which distinguishes it from all currently established genera within the family Geminiviridae [87]. Currently, studies have demonstrated that the V2 protein encoded by mulberry crinkle leaf virus acts as a replication enhancer by significantly increasing viral DNA accumulation both in planta and in protoplast systems, while the V3 protein of mulberry crinkle leaf virus functions as a viral suppressor of RNA silencing [88,89]. An isometric virus with a bipartite positive-sense RNA genome, tentatively named mulberry mosaic leaf roll-associated virus, has been identified as a putative new member of subgroup A within the genus Nepovirus, based on polyprotein phylogeny, CP sequence identity, and 3′-UTR characteristics [90]. Mulberry vein banding associated virus (MVBaV) is a distinct tospovirus characterized by a tripartite RNA genome encoding conserved replicative and structural proteins, and phylogenetically bridges two subgroups within the watermelon silver mottle virus serogroup [91]. Another mulberry MVBaV poses a significant threat to the sericulture industry due to its high incidence (66.7% in surveyed samples) in Guangxi Province and its potential for yield loss, compounded by the presence of thrips as suspected vectors [92]. Mulberry badnavirus 1 (MBV1) is a unique badnavirus characterized by a single open reading frame encoding conserved pararetroviral motifs, the co-encapsidation of full-length and deleted genomic forms that retain infectivity [93]. The mulberry crinivirus (MuCV), identified as a novel bipartite member of the genus Crinivirus (family Closteroviridae), features an RNA1 segment encoding key replicative domains and an RNA2 segment with conserved orthologous open reading frames [94,95]. Recently, a novel tri-segmented, negative-sense RNA virus was identified in mulberry and classified within the genus Rubodvirus, with its genome segments encoding an RNA-dependent RNA polymerase, a movement protein, and a nucleocapsid protein [96].

*MaNCED1* is primarily responsive to pathogen stress, and catalyzes the cleavage of 9-cis-violaxanthin to produce xanthoxin, thereby contributing to ABA precursor biosynthesis in mulberry [46]. *MaEXPA11* acts as a dual-functional regulator, positively enhancing mulberry resistance against *Ciboria shiraiana* infection while negatively modulating its tolerance to cold and drought stresses [97]. The HMLX56 protein, a chitin-binding protein isolated from mulberry, exhibits broad-spectrum resistance by demonstrating direct toxicity against *Plutella xylostella* (diamondback moth) and, when heterologously expressed in *Arabidopsis*, confers enhanced resistance to aphids, the fungal pathogen *Botrytis cinerea*, and the bacterial pathogen *Pseudomonas syringae* pv. tomato DC3000 [98]. The endophytic bacterium *Bacillus subtilis* Lu144, isolated from mulberry, demonstrates effective biocontrol activity against *Ralstonia solanacearum* by reducing bacterial wilt incidence through root colonization, systemic movement within intercellular spaces, and stable persistence in leaf tissues [99]. Eight mulberry germplasm accessions (BR-8, Karanjtoli-1, Hosur-C8, Nagalur Estate, Tippu, Calabresa, Thai Pecah, and SRDC-3) were identified as promising resistant sources, with most derived from mulberry, demonstrating potential for use in root-knot nematode-resistance breeding programs or as resistant rootstocks [100].

## 3. Medicinal Value of Mulberry

**Figure 2:**
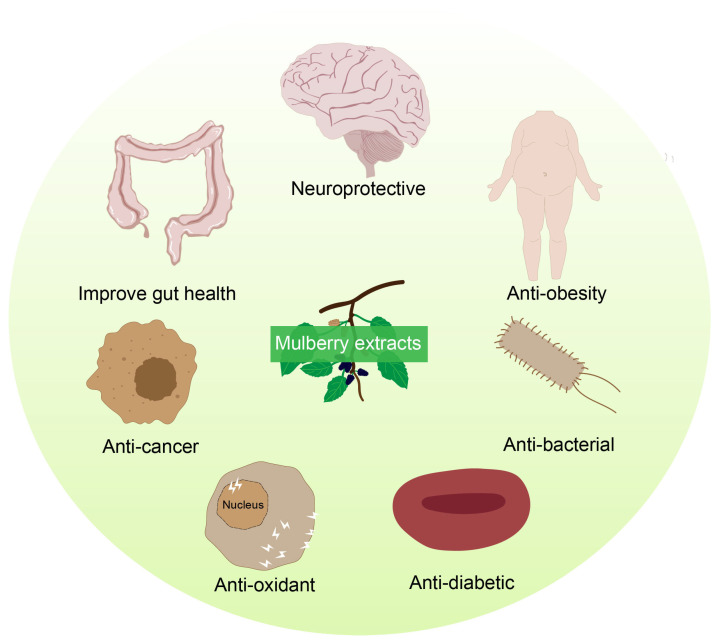
Schematic diagram summarizing the major health promoting effects of mulberry extracts. The pharmacological effects of mulberry extract include neuroprotection, such as ameliorating Parkinson’s disease and Alzheimer’s disease, as well as improving memory. Additionally, it promotes intestinal health, exhibits anticancer, antioxidant, and antibacterial activities, and contributes to lowering blood glucose levels and reducing obesity.

Mulberry, a perennial plant with a long history of use in traditional Chinese medicine, provides multiple medicinal parts including the leaves, twigs, root bark, and fruits [101,102]. Each part exhibits distinct pharmacological profiles and modern bioactivities, such as antioxidant, hypoglycemic and hyperlipidemia action, anti-inflammatory, anti-tumor, anti-cancer, anti-bacterial and anti-viral activity, hepatoprotective and renoprotective activities, anti-aging activity and so on [103,104,105]. China has been recognized as one of the earliest regions to domesticate silkworms and cultivate mulberry, with its documented use dating back thousands of years [106]. Historical traditional Chinese medicine classics, such as Shennong Ben Cao Jing, Tang Ben Cao, and Ben Cao Gang Mu, contain systematic records of its medicinal applications [101]. In recent decades, numerous cultivars and wild varieties of mulberry have been widely distributed and cultivated across China [107,108]. Owing to the abundance of bioactive compounds and their validated physiological effects, different parts of mulberry remain highly valued in both traditional and contemporary phytomedicine (Figure 2 and Table 1).

### 3.1. Phenolic Components

The 70% ethanol extract of mulberry fruit exhibits significant neuroprotective effects in both cellular and animal models of Parkinson’s disease, demonstrating dose-dependent prevention of dopaminergic neuronal loss through antioxidative and anti-apoptotic mechanisms, highlighting its potential therapeutic value for Parkinson’s disease intervention [109]. Mulberry fruit extract containing abundant flavonoid compounds enhances memory in mice by upregulating hippocampal nerve growth factor (NGF) release, which subsequently activates downstream signaling pathways, promotes synaptogenesis, cholinergic function, and neurogenesis, ultimately improving learning and memory performance [110]. Mulberry fruit extract ameliorates cytotoxicity and cognitive deficits in an Alzheimer’s disease mouse model by dissociating intracellular amyloid-β oligomers and restoring antioxidant activity [111]. Mulberry leaf polyphenol extracts (MLPE) counteract endoplasmic reticulum stress-induced resistance to doxorubicin in hepatocellular carcinoma cells, thereby restoring drug sensitivity and promoting caspase-3-dependent apoptosis [112]. MLPE also exhibits potential as an anticancer agent by modulating both autophagy and apoptosis in a p53-dependent manner. Furthermore, it elucidates the mechanistic role of p53 in mediating MLPE-induced cytotoxicity in hepatocellular carcinoma cells [113].

Anthocyanins, a subclass of water-soluble phenolic compounds within the flavonoid family, serve as natural pigments in plants [114]. To date, mulberry *MYB* genes, particularly those of the R2R3 subclass, encode functionally diversified transcription factors that regulate secondary cell wall biosynthesis, stress responses, and secondary metabolism, with specific members playing key roles in flavonoid biosynthesis and anthocyanin accumulation [115]. Mulberry fruits are known as rich sources of anthocyanins, containing at least sixteen identified anthocyanins, with cyanidin-3-O-glucoside and cyanidin-3-O-rutinoside as the predominant compounds [116]. Cyanidin-3-glucoside and cyanidin-3-rutinoside suppress the migration and invasion of highly metastatic A549 lung cancer cells [117]. Aqueous extracts of white mulberries possess pronounced antioxidant activity, which correlates with elevated levels of phenolic compounds and anthocyanins, alongside notable antibacterial efficacy against major enteric pathogens by suppressing bacterial proliferation and impeding their adhesion to intestinal epithelial cells, thereby supporting their promise as a functional food ingredient endowed with both antioxidant and antimicrobial attributes [118]. Another demonstrates that mulberroside A (MsA) exerts comprehensive neuroprotective effects in Alzheimer’s disease models by enhancing cholinergic function, reducing Aβ production via dual modulation of APP processing, attenuating oxidative stress and tau hyperphosphorylation through PI3K/AKT/GSK3β signaling, and upregulating neurotrophic pathways, highlighting its multi-target therapeutic potential [119].

Morusin, a prenylated flavone derived from mulberry bark, has attracted significant interest due to its diverse and potent biological properties [120]. Comprehensive studies have confirmed that morusin exerts marked analgesic, antioxidant, anti-inflammatory, osteogenic, antitumor, cardioprotective, neuroprotective, hepatoprotective, antidiabetic, and antimicrobial activities [121,122]. In details, morusin exerts anti-cancer effects in non-small cell lung cancer cells by inducing mitochondria-dependent apoptosis and promoting autophagy through modulation of ROS-mediated PI3K/AKT, JNK, and ERK signaling pathways [123]. Morusin inhibits hepatocellular carcinoma progression by inducing AMPK-mediated G1 cell cycle arrest and suppressing glycolysis through downregulation of key metabolic enzymes including Hexokinase 2, Pyruvate Kinase M2, and Lactate Dehydrogenase [124]. Another study showed that morusin exerts anti-hepatocellular carcinoma effects by directly targeting and inhibiting the ATP citrate lyase, thereby inducing ROS accumulation, PINK1/Parkin-mediated mitophagy, and mitochondrial apoptosis, highlighting its potential as a novel therapeutic candidate for hepatocellular carcinoma [125]. Morusin exhibits selective anti-breast cancer activity by inducing dose- and time-dependent apoptosis through downregulation of Survivin and upregulation of Bax, along with activation of caspase-3/9 pathways, while sparing normal breast epithelial cells [126].

Despite significant progress in antiretroviral therapy, the discovery and development of safe, efficacious, and multitargeted therapeutic agents against HIV continue to pose a formidable challenge. Mulberroside C, a bioactive compound derived from mulberry stem bark, demonstrates potent anti-HIV activity by functioning as a protease inhibitor [127]. Neochlorogenic acid, an active phenolic compound identified in Cortex Mori, demonstrates potent anti-HIV-1 activity by significantly suppressing reverse transcriptase products and potentially targeting key signaling pathways such as those mediated by haemopoietic cell kinase and epidermal growth factor receptor [128]. Moreover, Kuwanon G, isolated from mulberry, demonstrates potent bactericidal activity against major oral pathogens such as *Streptococcus mutans* and *Porphyromonas gingivalis*, mechanistically linked to the induction of cell wall damage and cytoplasmic condensation as visualized by transmission electron microscopy [129]. Moracin D derived from mulberry suppresses proliferation and induces apoptosis in breast cancer cells by inhibiting the Wnt3a/FOXM1/β-catenin signaling pathway while concurrently activating caspase cascades and glycogen synthase kinase 3β (GSK3β) [130]. Moracin N inhibits lung cancer tumorigenesis by directly targeting PD-L1 to disrupt the PD-1/PD-L1 interaction, thereby enhancing T cell-mediated anti-tumor immunity and synergizing with anti-PD-1 therapy in vivo [131]. Moracin N suppresses non-small-cell lung cancer by inducing mitochondrial apoptosis and promoting autophagic cell death via ROS accumulation and mTOR pathway inhibition [132]. Cudraflavone B (1) exhibits significant anti-inflammatory activity by inhibiting NF-κB translocation, thereby suppressing TNFα secretion and COX-2 expression, while demonstrating dual COX-1/COX-2 inhibition with higher selectivity for COX-2 than indomethacin [133]. Guangsangon E exhibits antitumor efficacy in both lung cancer and triple-negative breast cancer by inducing autophagy and mitochondrial dysfunction [134,135]. Sanggenon C suppresses lung cancer progression by selectively targeting and inhibiting SLC7A11 (implicated in the transport of cystine), thereby depleting glutathione synthesis, inactivating GPX4, and promoting the accumulation of lipid peroxides and reactive oxygen species to induce ferroptosis [136]. Sanggenol L exerts anti-prostate cancer effects through dual mechanisms: inducing both caspase-dependent and -independent apoptosis via suppression of the PI3K/AKT/mTOR pathway, and arresting cell cycle progression through p53 activation [137]. In addition, morusflavone exhibits significant binding affinity and stability with CYP17A1, suggesting its potential as a novel natural inhibitor for targeting CYP17A1 in prostate cancer therapy, meriting further preclinical and clinical investigation [138].

While accumulating evidence supports the pharmacological benefits of polyphenols for human health, significant attention must be paid to their actual bioactivity in vivo, particularly concerning the interplay between their chemical structures and key physiological processes such as intestinal absorption, metabolism, and overall bioavailability [139].

### 3.2. Alkaloids

1-Deoxynojirimycin (DNJ), a prominent piperidine alkaloid predominantly present in mulberry leaves, functions as a potent α-glucosidase inhibitor, thereby impairing disaccharide digestion and glucose absorption in animals [140]. DNJ has been identified as a key agent mediating anti-tumor, anti-viral, anti-inflammatory, anti-obesity, anti-diabetic effects in human and animal studies [141,142,143]. Evidence from other studies indicates that DNJ, as a potent α-glucosidase inhibitor derived from mulberry, has been proposed to mitigate hyperglycemia and potentially prevent the onset of diabetes mellitus [144]. Currently, studies have already discovered that transcriptome sequencing of mulberry leaves with varying DNJ levels enabled the identification and differential expression analysis of candidate genes, including lysine decarboxylase and primary-amine oxidase, which are proposed to be involved in the biosynthetic pathway of the DNJ [145,146]. Moreover, sangzhi alkaloids (SZ-A) ameliorate hypercholesterolemia in diabetic rats by modulating bile acid metabolism through gut microbiota restructuring and hepatic farnesoid X receptor (FXR) signaling regulation, thereby promoting cholesterol excretion [147]. Additionally, SZ-A exhibit anti-inflammatory and mucosal repair properties beneficial for ulcerative colitis treatment [148].

### 3.3. Polysaccharides

Polysaccharides from mulberry, particularly those derived from its medicinal and edible fruits and leaves, possess promising bioactivities encompassing antidiabetic, immunomodulatory, anti-inflammatory, antioxidant, anti-obesity, hepatoprotective, and renoprotective effects, supporting their therapeutic and sanitarian potential [149]. Mulberry leaf polysaccharides, particularly SY01-23 (rhamnose, glucuronic acid, galacturonic acid, glucose, galactose, xylose and arabinose), selectively modulate the gut microbiota by promoting the growth of beneficial Bacteroides species such as *Bacteroides ovatus* and *Bacteroides cellulosilyticus*, which in turn produce short-chain fatty acids including acetate and propionate, thereby contributing to improved gut health and overall host wellness [150].

**Table 1:** Medicinal value of mulberry extract.

Category	Name	Medicinal Value	Subject	References
Phenolic Components	Mulberry fruit extract	Neuroprotective effects Improve memory Parkinson’s disease Alzheimer’s disease	Mouse	[109,110,111]
	Mulberry leaf polyphenol extracts	Anti-cancer	Human Hepatoma HepG2 Cells	[112,113]
	Anthocyanins	Anti-lung cancer Anti-oxidant activity Anti-bacterial	Human lung cancer cells Bacteria	[117,118]
	Mulberroside A (MsA)	Anti-oxidant activity Anti-bacterial	Mouse	[119]
	Morusin	Analgesic Anti-oxidant Anti-inflammatory Osteogenic Anti-tumor Cardioprotective Neuroprotective Hepatoprotective Anti-diabetic Anti-microbial activities	Human lung cancer cells Human hepatocellular carcinoma cells Human breast epithelial cells	[123,124,125,126]
	Mulberroside C	Anti-HIV	In vitro experiments	[127]
	Neochlorogenic acid	Anti-HIV	Human 786-O cells	[128]
	Kuwanon G	Anti-bacterial	In vitro experiments	[129]
	Moracin D	Anti-breast cancer	Human breast cancer cells	[130]
	Moracin N	Anti-lung cancer	Human lung cancer cells	[131,132]
	Cudraflavone B (1)	Anti-inflammatory	human monocyte cells	[133]
	Guangsangon E	Anti-breast cancer Anti-lung cancer Anti-nasopharyngeal cancer	Human breast cancer cells Human A549 and CNE1 cells	[134,135]
	Sanggenon C	Anti-lung cancer	Human lung cancer cells	[136]
	Sanggenol L	Anti-prostate cancer	Human prostate cancer cells	[137]
	Morusflavone	Anti-prostate cancer	In vitro experiments	[138]
Alkaloids	1-Deoxynojirimycin (DNJ)	Anti-tumor Anti-viral Anti-inflammatory Anti-obesity Anti-diabetic	Mouse Human	[142,143,144]
	Sangzhi alkaloids (SZ-A)	Anti-diabetic Anti-inflammatory Ameliorated hypercholesterolemia	Mouse	[147,148]
Polysaccharides	Mulberry leaf polysaccharides	Improve gut health	In vitro experiments	[150]

## 4. From Defense to Therapy: Enhancing Stress Tolerance and Medicinal Quality in Mulberry

Metabolomic analysis revealed that drought stress substantially activated the flavonoid biosynthesis pathway in mulberry, as indicated by the significant accumulation of upstream precursors and pathway intermediates, including various phenolic acids [151]. Integrative metabolomic and transcriptomic analyses reveal that salt stress actively promotes flavonoid accumulation in germinating mulberry seeds by modulating the expression of key enzymes in the phenylpropanoid and flavonoid pathways, highlighting a concentration-dependent regulatory mechanism [152]. Metabolomic profiling reveals that manganese stress actively reprograms secondary metabolism in mulberry, inducing a suite of differentially expressed metabolites (including terpenoids and phenolics) that are significantly enriched in pathways like alpha-linolenic acid metabolism, thereby contributing to Mn tolerance [74]. Boron stress (deficiency or toxicity) in mulberry significantly activates the biosynthesis of diverse secondary metabolites, which serve as key adaptive components in the plant’s physiological and metabolic defense network, particularly involving pathways related to amino acid metabolism and the biosynthesis of other secondary metabolites [153]. Mechanical damage and herbivory increase the release of secondary metabolites from mulberry leaves [154]. Integrated proteomic and metabolomic analyses reveal that UV-B and *Botrytis cinerea* stresses commonly enhance the flux through both the flavonoid and lignin biosynthesis branches in mulberry [155]. Chalcomoracin, a prominent secondary metabolite derived from mulberry leaves infected by fungi, has been identified as a potent anticancer agent, emerging as a promising candidate for cancer therapy [156].

**Figure 3:**
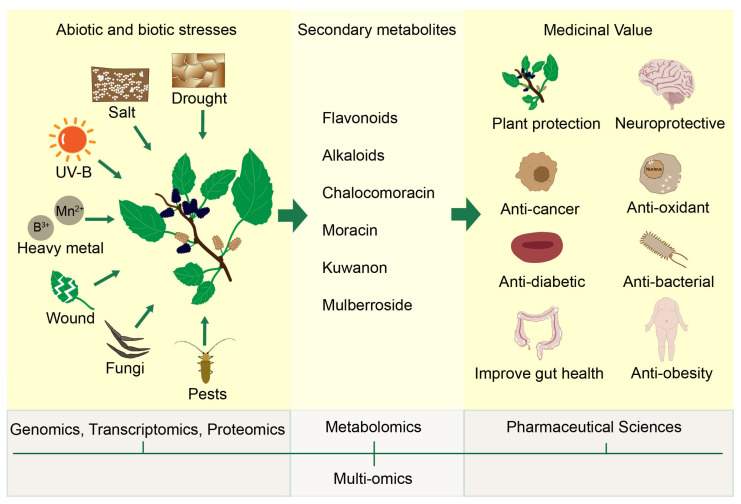
Schematic diagram depicting the correlation between abiotic/biotic stresses and the accumulation of bioactive secondary metabolites in mulberry. Established research demonstrates that various stresses (e.g., drought, salinity, UV-B, heavy metals, wounding, fungal infection, and herbivory) promote the biosynthesis of secondary metabolites. These compounds play a dual role: they are crucial for mulberry’s environmental stress adaptation and underpin the medicinal value of mulberry extracts for disease therapy.

In summary, accumulating evidence demonstrates that both abiotic and biotic stresses consistently activate the biosynthesis of diverse secondary metabolites in mulberry. These multi-omics analyses reveal that stresses enhance metabolic flux through key pathways, including phenylpropanoid, flavonoid, lignin, terpenoid, and phenolic branches, leading to the accumulation of stress-specific compounds such as chalcomoracin and various phenolic acids. This stress-induced metabolic reprogramming not only serves as a core adaptive strategy for enhancing stress tolerance but also presents a strategic opportunity to elevate the concentration of pharmaceutically valuable compounds in mulberry. By leveraging controlled stress application or elucidating the underlying regulatory mechanisms, it is possible to develop targeted cultivation or biotechnological approaches that simultaneously improve plant resilience and enrich the medicinal quality of mulberry-derived materials (Figure 3).

## 5. Perspectives

Mulberry plays a pivotal role in the sericulture industry and possesses significant medicinal value. The expansion of the sericulture sector has broadened markets for mulberry, its by-products, and associated silkworm derivatives, contributing to increased farmer income and employment. However, research into the genetic basis of stress tolerance and disease resistance in mulberry lags significantly behind that of model plants like *Arabidopsis* and staple crops such as wheat, rice, and maize. This knowledge gap impedes the application of molecular breeding for cultivar improvement. To address these challenges, this review investigates the responses of mulberry to both abiotic and biotic stresses, aiming to identify genetic targets for enhancing stress tolerance through molecular breeding. Concurrently, harnessing controlled environmental stress to stimulate secondary metabolite biosynthesis offers a strategy to improve the medicinal properties of mulberry-derived products. Future work should prioritize the integration of multi-omics technologies—such as genomics, transcriptomics, proteomics, and metabolomics—to discover and characterize key genes governing stress tolerance, disease resistance, and the biosynthesis of pharmaceutically valuable compounds in mulberry.

## Data Availability

No new data were created or analyzed in this study. Data sharing is not applicable to this article.

## Abbreviations

AKT	Protein Kinase B found from AKT8 strain
APX	Ascorbate Peroxidase
CAT	Catalase
ERK	Extracellular Signal-Regulated Kinase
FOXM1	Forkhead Box M1
JNK	c-Jun N-terminal Kinase
mTOR	Mammalian Target of Rapamycin
NCED1	9-cis-Epoxycarotenoid Dioxygenase 1
NF-κB	Nuclear Factor Kappa-B
PCS	Phytochelatin synthase
PD-L1	Programmed Death-Ligand 1
PI3K	Phosphatidylinositol 3-kinase
PINK1	PTEN-induced putative kinase 1
POD	Peroxidase
ROS	Reactive oxygen species
SOD	Superoxide Dismutase
TCA cycle	Tricarboxylic Acid Cycle
TNFα	Tumor Necrosis Factor α
UV-B	Ultraviolet B
Wnt3a	Wingless-type MMTV integration site family, member 3a
